# Treating post-traumatic osteomyelitis of a metacarpal fracture with the ‘mini-masquelet’ procedure

**DOI:** 10.1016/j.tcr.2023.100945

**Published:** 2023-09-25

**Authors:** Lydian Huisman, Eelke Bosma

**Affiliations:** a, Dept. of Surgery, University Medical Center Groningen, Hanzeplein 1, 9713GZ Groningen, the Netherlands; b Dept. of Surgery, Martini Ziekenhuis Groningen, Van Swietenplein 1, 9728NT Groningen, the Netherlands

**Keywords:** Osteomyelitis, Fracture, Metacarpus, Masquelet

## Abstract

**Background:**

Post-traumatic osteomyelitis is a challenging complication after a fracture, requiring long-term treatment to prevent loss of function. One treatment strategy is the biphasic masquelet technique, focussing on both control of the infection and bone reconstruction. This technique is mainly used to treat defects of the long bones. Very little literature exists about the masquelet procedure for treatment of defects of smaller bones. We describe a case of post-traumatic osteomyelitis after a metacarpal fracture, treated with the ‘mini-masquelet’ technique.

**Patient case:**

A 23-year old woman was treated with the masquelet procedure for osteomyelitis and bone loss following a metacarpal IV fracture of her right hand. After 29 weeks, she had full range of motion of both the hand and fingers.

**Conclusion:**

The ‘mini-masquelet’ technique as a strategy to treat osteomyelitis and reconstruct bone loss after a metacarpal fracture, can reduce potential loss of function and loss of quality of life. This technique appears to be widely applicable for treatment of complex hand injuries and osteomyelitis of the hand.

## Introduction

Post-traumatic osteomyelitis after a fracture is a challenging complication, occurring in 1–2 % of fractures of the extremities. It often requires long-term treatment to prevent bone loss and/or impaired healing [1]. Treatment is based on both infection control and bone reconstruction. The so-called masquelet procedure is a two-phase procedure based on these principles. In the first phase, after debridement, a biomembrane is created using an (antibiotic-containing) cement spacer to bridge the bone defect. In the second phase, the spacer is removed and the remaining defect is filled with (autologous) cancellous bone [2]. This method is mainly used for defects of the long bones. Literature on this procedure as a treatment for smaller bones is limited. In this article, we describe the use of this method for post-traumatic osteomyelitis with bone loss after a metacarpal fracture: the ‘mini-masquelet’.

## Patient case

A 23-year-old woman presented to the Emergency Department after a fall with displaced shaft fractures of the fourth and fifth metacarpal bones of her right hand (Fig. A), for which she underwent plate osteosynthesis (Fig. B). Five weeks postoperatively, she presented with signs of wound infection. Operative exploration revealed a deep infection with osteomyelitis of the fourth metacarpal bone. After radical debridement and irrigation, a gentamicin sponge was left in place. A loose screw was removed, but the plate was left in situ for stability. Antibiotics were started according to protocol. The osteosynthesis material of the fifth metacarpal bone was left untouched.

**Fig. A:**
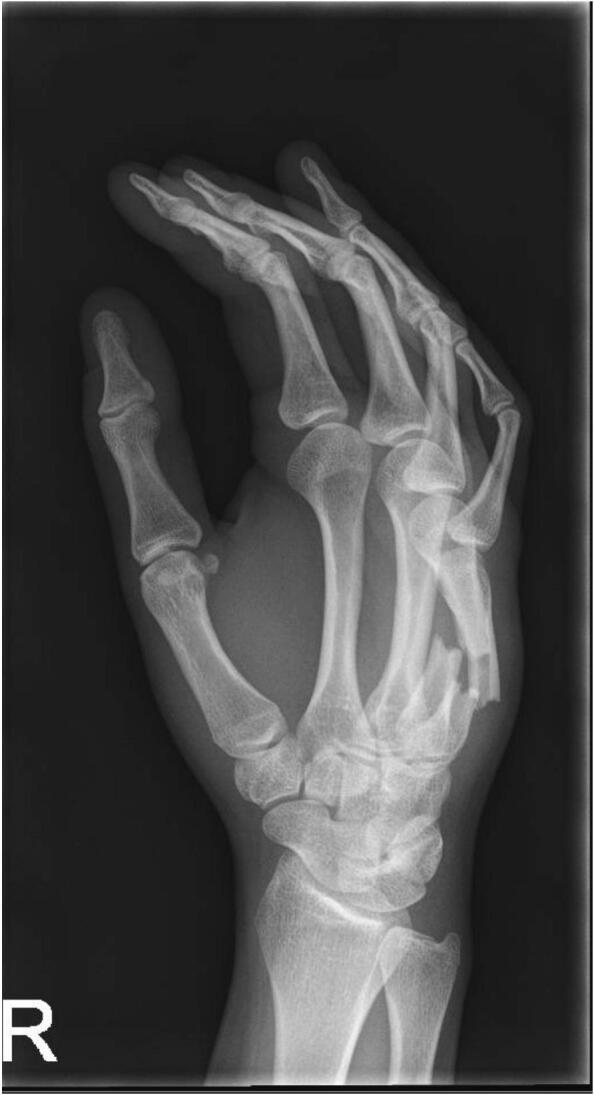
Displaced midshaft fractures of metacarpals IV and V at initial presentation on the Emergency Department, shown on a three-quarter radiograph view.

**Fig. B:**
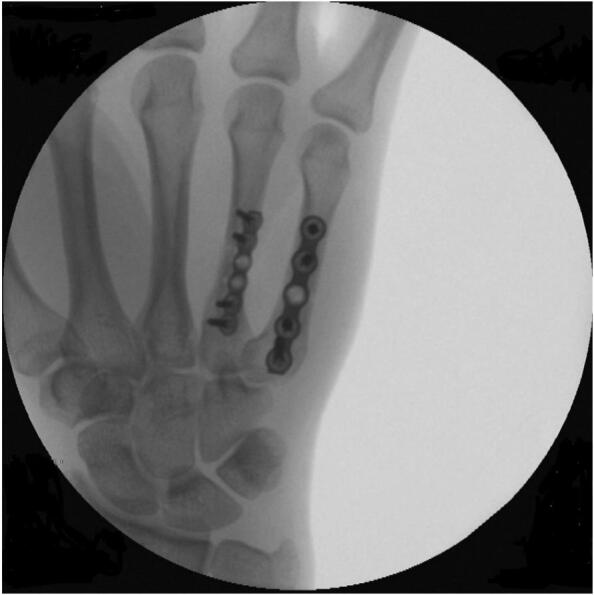
Intraoperative images after plate osteosynthesis of both fractures.

A week later, the gentamicin sponge was removed. Cultures were taken and irrigation was performed, with no signs of infection. The plate on the fourth metacarpal bone was removed. The distal and proximal fragments were fixed outside the wound area with K-wires. A vancomycin-coated cement spacer was placed in the remaining defect of the fourth metacarpal bone (Fig. C).

**Fig. C:**
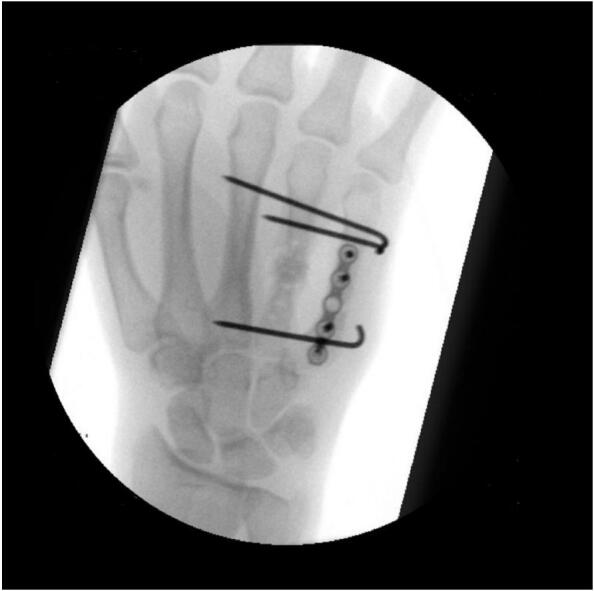
Intraoperative images after removal of the plate and screws from metacarpal IV, with cement spacer in place, stabilized using three Kirschner-wires (first phase of the Masquelet procedure).

After six weeks, the spacer and K-wires were removed. The remaining defect of metacarpal IV was filled with autologous bone graft as the second phase of the procedure, followed by osteosynthesis. Due to stiffness in the metacarpophalangeal joints (MCPs), tenolysis of the extensor digitorum communis of the fourth and fifth rays was performed. Passive flexion of 90 degrees in the MCPs was obtained during surgery. Eight weeks later, a hand radiograph showed no consolidation of metacarpal IV, without signs of complications. A bone growth stimulator was started, and after another eight weeks, metacarpal IV was consolidated (fig. D). However, there was still a flexion limitation of MCP IV and V of 30 degrees. The osteosynthesis material was removed and tenolysis and arthrolysis of the fourth and fifth rays were performed. After this, flexion of MCP IV and V was fully restored. Again seven weeks later, she had full hand function, including full flexion and extension of both the MCPs, proximal interphalangeal and distal interphalangeal joints.

**Fig. D:**
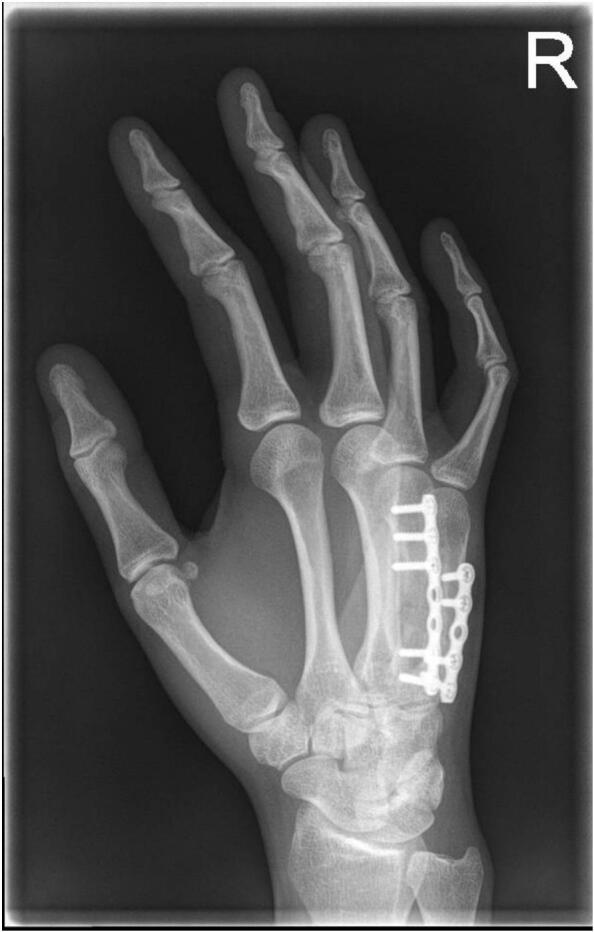
Radiograph at sixteen weeks post-iliac cancellous bone grafting (second phase of the Masquelet procedure) and plate osteosynthesis. Nearly complete consolidation of metacarpal IV was achieved.

## Considerations

Post-traumatic osteomyelitis with bone loss is relatively rare. The two-stage masquelet procedure is an important treatment strategy, which has been limitedly described for these defects in small bones. The procedure was described, however, as treatment for bone defects as a result of trauma, using a spacer without antibiotics. Our case illustrates the use of this strategy for osteomyelitis with bone loss after a metacarpal fracture, with full recovery of hand function.

Previously, a case series was reported of seven patients who underwent the masquelet procedure for osteomyelitis of phalanges of various etiologies [3]. In all patients, a cement spacer was placed after radical debridement and removed after four weeks to facilitate rapid rehabilitation. The defects were filled with cancellous bone and stabilized with osteosynthesis material. After two to three months, complete bone healing was achieved in all cases. Additionally, a case was described of a patient treated with the masquelet technique, with a metacarpal I fracture and osteomyelitis secondary to surgical fracture treatment [4]. The cement spacer was removed after eight weeks; the remaining defect filled with cancellous bone and stabilized with a plate. Three months later, the bone was completely healed, and hand function was fully restored.

With our patient, we used an antibiotic-coated spacer. This had the advantage of delivering a high local dose with a smaller chance of side effects, as compared to systemic antibiotics. There is no consensus regarding the necessity of systemic antibiotic treatment after extensive debridement of the infected area, alongside local treatment with the coated cement spacer. The typical timing for removing the spacer usually falls between six and eight weeks to promote soft tissue healing and optimize the effectiveness of the antibiotic [5]. However, human and animal research suggests that the optimal timing is around four weeks due to the more favorable biochemical status of the membrane at that time. This could enhance osteogenesis [6,7].

## Conclusion

With our case, we illustrate the outcomes of this procedure in osteomyelitis with bone loss after a metacarpal fracture, with complete functional recovery. The application of the “mini-masquelet” for osteomyelitis and subsequent bone loss of the hand can thus potentially prevent or reduce functional loss and loss of quality of life. Unlike other reconstruction techniques, such as transplantation of vascularized bone, the masquelet procedure can be performed in infected areas and in acute settings [2]. Although widespread use in hand surgery has not yet been confirmed, the procedure seems widely applicable in complex hand injuries and (posttraumatic) osteomyelitis of the bones of the hand.

## Declaration of competing interest

Both authors declare no potential conflicts of interest with respect to the research, authorship, and/or publication of this article. The authors received no financial support for the research, authorship, and/or publication of this article.
